# Case Report: Adult Still’s Disease in an Alemtuzumab-Treated Multiple Sclerosis Patient

**DOI:** 10.3389/fimmu.2020.02099

**Published:** 2020-08-28

**Authors:** Julia Krämer, Tanja Krömer-Olbrisch, Heinz-Jürgen Lakomek, Peter D. Schellinger, Dirk Foell, Sven G. Meuth, Vera Straeten

**Affiliations:** ^1^Department of Neurology with Institute of Translational Neurology, University Hospital Münster, Münster, Germany; ^2^Department of Dermatology, Venerology, Allergology, and Phlebology, Johannes-Wesling-Hospital Minden, University Hospital Ruhr University of Bochum, Minden, Germany; ^3^Department of Rheumatology and Physical Medicine, Johannes-Wesling-Hospital Minden, University Hospital Ruhr University of Bochum, Minden, Germany; ^4^Department of Neurology and Neurogeriatrics, Johannes-Wesling-Hospital Minden, University Hospital Ruhr University of Bochum, Minden, Germany; ^5^Department of Pediatric Rheumatology and Immunology, University Hospital Münster, Münster, Germany

**Keywords:** multiple sclerosis, Adult onset still disease, secondary autoimmunity, alemtuzumab, rituximab, anakinra, case report

## Abstract

**Background:**

Autoimmune adverse events are the most relevant risks of alemtuzumab therapy. We present a patient with relapsing-remitting multiple sclerosis, who developed adult-onset Still’s disease (AOSD) following alemtuzumab treatment.

**Case Presentation:**

The patient suffered from sore throat, swallowing difficulties, high spiking quotidian fever, generalized skin rash, arthritis, and myalgia 2 months after the second course of alemtuzumab. Laboratory tests revealed elevated acute-phase reactants, anemia, neutrophilic leukocytosis, and thrombocytosis. Serum calprotectin, interleukin-2, and interleukin-6 levels were strongly increased. Autoimmune, rheumatic, neoplastic, infectious, and granulomatous disorders were excluded. The NLRP1 and NLRP3 gene test, which was performed under the presumption of a cryopyrin-associated autoinflammatory syndrome, was negative. Based on the Yamaguchi and Fautrel criteria, and supported by the histological findings from a skin biopsy of the rash, the diagnosis of AOSD was established. Therapy with the anti-IL-1 agent (anakinra) led to a significant improvement of symptoms and blood parameters. However, anakinra had to be converted to rituximab due to generalized drug eruption. Following therapy with rituximab, the patient has fully recovered.

**Conclusion:**

The current case highlights AOSD as another rare and potentially life-threatening secondary autoinflammatory/autoimmune event following alemtuzumab treatment.

## Introduction

The use of alemtuzumab as therapy for active relapsing-remitting multiple sclerosis was recently restricted due to safety concerns. The potential development of secondary autoimmunity represents the most relevant risk of this therapy (1). In the last 2 years, previously unknown autoimmune phenomena, such as sarcoidosis, vitiligo, autoimmune hepatitis, hemophagocytic lymphohistiocytosis, and idiopathic multicentric Castleman’s disease were described in real-world cohorts (2–4). Here, we present for the first time a case of adult-onset Still’s disease (AOSD) following alemtuzumab treatment.

## AOSD

Adult-onset Still’s disease is a rare, multigenic, clinically highly variable, multi-systemic inflammatory disease of unknown etiology, and pathogenesis usually affecting young adults. It is typically characterized by high spiking fever, arthralgia or arthritis, and transient maculopapular salmon-pink evanescent skin rash. Other manifestations are sore throat or pharyngitis, dynophagia, myalgia or myositis, generalized lymphadenopathy, hepatosplenomegaly, serositis, and cardiopulmonary or kidney involvement (5, 6). Common laboratory abnormalities include neutrophilic leukocytosis, elevated acute-phase reactants, inflammatory anemia, and thrombocytosis accompanied by liver abnormalities. The diagnosis is primarily clinical, necessitates the exclusion of a wide range of mimicking disorders, and still lacks specific diagnostic testing (5, 6). Different diagnostic criteria have been proposed for AOSD diagnosis (Supplementary Table S1). Therapy remains essentially empirical comprising traditionally non-steroidal anti-inflammatory drugs, corticosteroids, and immunosuppressants. Recently biological therapies such as tumor necrosis factor-α inhibitors, and IL-1, -18, and -6 antagonists (5, 6) were introduced as potential therapies for AOSD patients.

## Case Report

### Patient Information

**FIGURE 1:**
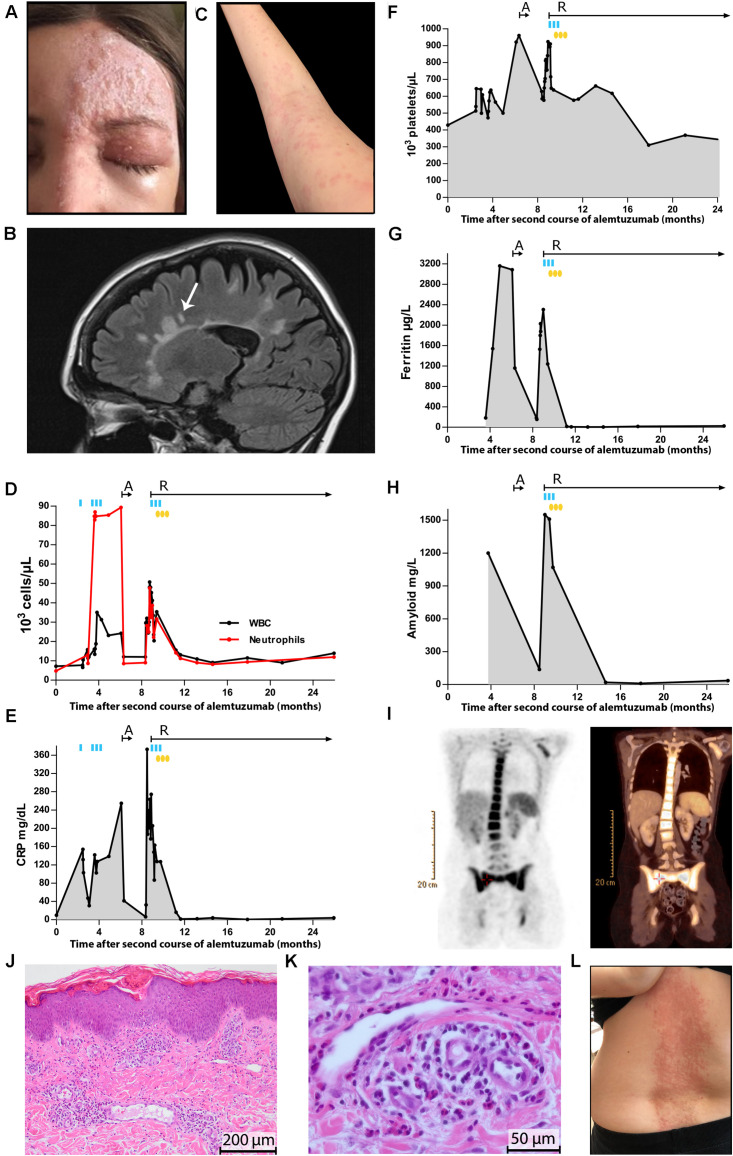
Diagnostic work-up and treatment regimes in an alemtuzumab-treated patient with relapsing-remitting multiple sclerosis developing AOSD. **(A)** Varicella-zoster virus-induced left ophthalmic zoster. **(B)** Sagittal fluid-attenuated inversion recovery of the brain demonstrating multiple non-enhancing T2 hyperintense lesions. One new, non-enhancing lesion is indicated by an arrow. **(C)** Evanescent maculopapular, salmon-pink skin rash on the right arm. Charts show the course of white blood cell counts and neutrophils **(D)**, CRP **(E)**, platelets **(F)**, ferritin **(G)**, and amyloid **(H)**. Time points and length of different treatment regimens are outlined 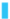: single administration of IVMPS; 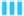: three doses of IVMPS on three consecutive days; 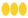: three doses of intravenous immunoglobulins on three consecutive days; arrows indicate the duration of therapies with anakinra (A) and rituximab (R). **(I)** FDG-PET/CT showing significant hypermetabolism in bone marrow and spleen. Hematoxylin and eosin staining of skin biopsy displaying periadnexal and perivascular infiltrates of inflammatory cells, surrounding superficial blood vessels, hair follicles, and the interstitium (in between the vessels and adnexal structures). Original magnification at 200 μm **(J)**. Hematoxylin and eosin stain showing perivascular inflammatory infiltration of lymphocytes, neutrophils, and eosinophils. Original magnification at 50 μm **(K)**. Generalized drug eruption on the back, chest area, arms, and legs due to therapy with anakinra **(L)**.

A 26-year-old, previously completely healthy, female Caucasian was first diagnosed with relapsing-remitting multiple sclerosis in August 2016. Family history of neurological and autoimmune diseases was negative. Due to high clinical disease activity (three relapses in 2016), she received two courses of alemtuzumab (10/2016 and 10/2017) as first-line therapy. Urticaria after the first course of alemtuzumab had to be treated with antihistamines. In February 2017, the patient suffered from varicella-zoster virus-induced left ophthalmic zoster (Figure 1A), which was treated with intravenous aciclovir. She developed pronounced postherpetic neuralgia, which was treated with pregabalin, opiods, and amitriptyline. The patient also developed Hashimoto’s disease 11 months after alemtuzumab initiation. Disease activity stabilized clinically over time, but cranial magnetic resonance imaging from April and October 2017 showed three new, non-enhancing T2 hyperintense lesions in the left operculum and periventricular (Figure 1B). 2 months after the second course of alemtuzumab, the patient presented to her local ear, nose, and throat (ENT) clinic with a sore throat and swallowing difficulties.

### Clinical Findings

The patient further suffered from high spiking quotidian fever of up to 40 degrees occurring in the evenings, and a generalized itching, evanescent maculopapular, salmon-pink skin rash (Figure 1C) that accompanied the fever. Pharyngitis was diagnosed and treated with antibiotics. Under single dose of intravenous methylprednisolone (IVMPS, Figures 1D,E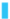) and antihistamines, the rash improved only temporarily. However, the patient developed arthritis and myalgia. She was referred to her local clinic of neurology, rheumatology, and dermatology.

### Diagnostic Assessment

Repeated dermatological examinations (radioallergosorbent test and total IgE) were not indicative. She developed anemia, neutrophilic leukocytosis, and thrombocytosis, and showed elevated acute-phase reactants (Figures 1D–F) including amyloid, interleukin-2 (1654 U/ml; reference value < 623 U/ml), and interleukin-6 levels (61.8 pg/ml; reference value < 7 pg/ml). Autoimmune, rheumatic, neoplastic, infectious, and granulomatous disorders were excluded as possible causes by repeated serological testing, blood and urine cultures, and imaging tests (chest x-ray, abdomen sonography, and transesophageal echocardiography). The NLRP1 and NLRP3 gene test, which was performed under the presumption of a cryopyrin-associated autoinflammatory syndrome, was negative.

### Treatment and Outcome

Following a probatory IVMPS therapy (three doses; Figures 1D,E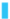) and subsequent oral prednisolone with slow dose tapering, symptoms improved, and inflammatory blood parameters decreased. When trying to reduce oral prednisolone, the symptoms came back, and inflammatory blood parameters and platelets immediately increased (Figures 1D–G). Serum calprotectin (157100 ng/ml; reference value < 2940 ng/ml) (7) and interleukin-2 levels (2129 U/ml; reference value < 623 U/ml) were strongly increased.

Based on the Yamaguchi and Fautrel criteria, and supported by the histological findings from a skin biopsy of the rash (Figures 1J,K) the diagnosis of AOSD was established (8, 9).

Therapy with the anti-IL-1 agent (anakinra), initiated in April 2018, led to a significant improvement of symptoms and blood parameters (Figures 1D–H). Besides anakinra, the patient was treated with oral prednisolone. However, anakinra had to be discontinued after 4 weeks due to generalized itching and follicular drug eruption (Figure 1L). Treatment was symptomatic, consisting of anti-H1 antihistamines. Whole-body ^18^F-FDG-PET/CT demonstrated significant hypermetabolism in bone marrow and spleen (Figure 1I).

Therapy termination led to a massive deterioration of symptoms and anemia, and an increase of acute-phase reactants, leukocytes, neutrophils, ferritin and amyloid (Figures 1D–H). Repeated IVMPS and oral prednisolone with slow dose tapering, intravenous immunoglobulins (Figures 1D–H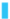,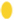), and initiation of rituximab therapy (Figures 1D–H) induced ongoing remission. The patient fully recovered and continues to receive a small dose of oral prednisolone and, every 6 months, rituximab. Therapy with hydroxychloroquine sulfate had to be discontinued due to hair loss. This adverse event disappeared immediately after termination of therapy. The patient is regularly examined at her neurological and rheumatological clinic. Serological testing is performed regularly. Since January 2019, the patient is working works fulltime again. However, 24 months after the second course of alemtuzumab, she developed a steroid-induced cataract of the left eye, which required an surgical intervention.

## Discussion

A strict risk management strategy and several tools implemented in real-world clinical settings, were developed for early detection and management of autoimmune events in patients treated with alemtuzumab (10). However, a variety of new rare autoimmune entities have been described in the past 2 years (2–4). The current case highlights AOSD as another rare and potentially life-threatening secondary autoinflammatory/autoimmune event following alemtuzumab treatment. The European Medicines Agency (EMA) has recently restricted the use of alemtuzumab as therapy for active relapsing-remitting MS due to reports of rare but serious and partially fatal side effects. Alemtuzumab should only be started in adults with relapsing-remitting MS that is highly active despite treatment with at least one disease-modifying therapy or if the disease is rapidly worsening (11). These safety issues, in the context of the increasing number of highly effective alternative disease-modifying therapies, makes alemtuzumab a less likely early treatment option to consider (12).

Even if the pathogenesis of the disease has not been elucidated in its entirety, activation of macrophages and neutrophils and subsequent proinflammatory cytokine storms triggered by activated immune cells play a pivotal role (6, 13). In terms of genetic susceptibility, several human leukocyte antigens (HLAs) have been reported to be associated with AOSD including *HLA-Bw35*, *-DRw6*, *HLA-DR4*, *HLA-B17*, *HLA-B18*, *HLA-B35*, and *HLA-DRB1* (6, 13). Interestingly, our patient expressed the *HLA-DRB1* allele that both increase the risk for MS and AOSD (6, 13). Moreover, the current case demonstrates a compatible response of AOSD to two different biological therapies: first, the anti-IL-1 agent anakinra (used for treating inflammatory diseases) and second, the monoclonal anti-CD20 antibody rituximab (used for treating autoimmune diseases). While anakinra is the standard therapy for AOSD, particularly in prednisone-refractory disease courses, rituximab was shown to be effective in treating AOSD, and also juvenile idiopathic arthritis, in numerous case studies (14, 15). This applies especially to patients in whom a strong activation of the adaptive immune system is essential for disease progress.

We consider the cutaneous adverse reaction after therapy with anakinra (Figure 1L) rather as generalized drug eruption than as drug rash with eosinophilia and systemic symptoms (DRESS) syndrome. DRESS syndrome is a rare, severe multiorgan and potentially fatal systemic hypersensitivity reaction mostly caused by a limited number of eliciting drugs, especially anti-convulsants and antibiotics (16–20). A DRESS syndrome was also described in a child following treatment with anakinra (20). In our case several facts speak against a DRESS syndrome: after initiation of anakinra our patient did not suffer from systemic symptoms (fever, rigors, and hypotension), lymphadenopathy, facial swelling or hematological abnormalities such as atypical lymphocytosis, thrombocytopenia, agranulocytosis, or eosinophilia. Involvement of visceral organs was excluded by whole-body ^18^F-FDG-PET/CT (Figure 1I). Infectious disorders (HHV-6, HHV-7, CMV, EBV, and VZV) were formally excluded as further possible causes.

Although an early and accurate diagnosis may lead to better outcomes, diagnosing AOSD is often difficult and typically delayed – as in our case – due to the presence of several non-specific symptoms and the absence of characteristic serological biomarkers. Thus, it took 4 months to establish the diagnosis of AOSD and initiate a suitable therapy with the anti-IL-1 blocker (anakinra).

The current case highlights AOSD as another potentially life-threatening secondary autoinflammatory/autoimmune event following alemtuzumab treatment. Thorough clinical follow-up and early intense interdisciplinary communication and intervention are necessary in suspicious cases after treatment with alemtuzumab.

## Patient Perspective

While treatment with corticosteroids caused only a short-term improvement, initiation of rituximab therapy induced long-lasting remission. Now, i am feeling fine again, but these 4 months were the worst experiences of my life. I do not want anyone to go through what I had to. In the end I hope that physicians all over the world have learned something from reading my case.

## Data Availability Statement

The raw data supporting the conclusions of this article will be made available by the authors, without undue reservation.

## Ethics Statement

Written informed consent to publish the present case details (clinical, histopathological, and imaging data and laboratory findings) was obtained from the patient. Written informed consent was obtained from the individual(s) for the publication of any potentially identifiable images or data included in this article.

## Author Contributions

JK, SM, and VS conceived the study and defined the concept. JK, TK-O, H-JL, PS, DF, SM, and VS collected and interpreted the data. JK wrote the initial draft of the manuscript. TK-O prepared the histopathological images. JK, TK-O, H-JL, PS, DF, SM, and VS critically discussed the data, revised the manuscript for intellectual content, and approved the version to be published. All authors agreed to be accountable for all aspects of the work in ensuring that questions related to the accuracy or integrity of any part of the work are appropriately investigated and resolved.

## Conflict of Interest

JK received honoraria for lecturing from Biogen, Novartis, Genzyme, Merck, Mylan, and Teva, and financial research support from Sanofi Genzyme and Novartis. TK-O, H-JL, and PS declare that the research was conducted in the absence of any commercial or financial relationships that could be construed as a potential conflict of interest. DF received honoraria for lecturing and travel expenses for attending meetings from Chugai-Roche, Novartis, and SOBI, and financial research support from Novartis, Pfizer, and SOBI. SM received honoraria for lecturing and travel expenses for attending meetings from Almirall, Amicus Therapeutics Germany, Bayer Health Care, Biogen, Celgene, Diamed, Genzyme, MedDay Pharmaceuticals, Merck Serono, Novartis, Novo Nordisk, ONO Pharma, Roche, Sanofi-Aventis, Chugai Pharma, QuintilesIMS, and Teva. His research is funded by the German Ministry for Education and Research (BMBF), Deutsche Forschungsgemeinschaft (DFG), Else Kröner Fresenius Foundation, German Academic Exchange Service, Hertie Foundation, Interdisciplinary Center for Clinical Studies (IZKF) Münster, German Foundation Neurology, and by Almirall, Amicus Therapeutics Germany, Biogen, Diamed, Fresenius Medical Care, Genzyme, Merck Serono, Novartis, ONO Pharma, Roche, and Teva. VS received honoraria for serving on Scientific Advisory Boards, lecturing and travel expenses for attending meetings from Novartis, Biogen, Merck Serono, Sanofi Genzyme, Roche, and Teva.

## Abbreviations

AOSD: adult-onset Still’s disease
DRESS: drug rash with eosinophilia and systemic symptoms
EMA: European Medicines Agency
ENT: ear, nose, and throat
HLA: human leukocyte antigen
IVMPS: intravenous methylprednisolone.
