# Supplementary material for: Case Report: Adult Still’s Disease in an Alemtuzumab-Treated Multiple Sclerosis Patient
Source: Front Immunol. 2020 Aug 28;11:2099. doi: 10.3389/fimmu.2020.02099 (PMC7493652; doi:10.3389/fimmu.2020.02099)
Supplement: Supplementary file 1 [file Table_1.DOCX]

| **Table S1** Criteria of AOSD. | | |
| --- | --- | --- |
| **Cush criteria^1^** | **Yamaguchi criteria^2^** | **Fautrel criteria^3^** |
| Probable AOSD: 10 points during 12 weeks observation  Definite AOSD: 10 points during 6 months of observation | ≥ 5 criteria including at least 2 major criteria  Exclusion criteria: infections, malignancies, rheumatic diseases | 4 major criteria, or 3 major and 2 minor criteria |
| **2 points each:**  Quotidian fever > 39°C  Transient rash  WBC > 12000/ml and ESR > 40 mm/h  Negative ANA/RF  Carpal ankylosis | **Major criteria:**  Fever ≥ 39 °C, intermittent, lasting ≥ 1 week  Arthralgia or arthritis, lasting ≥ 2 week  Typical rash  WBC > 10000/ml (> 80% Neutrophil granulocytes) | **Major criteria:**  Spiking fever ≥ 39 °C  Arthralgia  Transient rash  Pharyngitis  Neutrophil granulocytes ≥ 80%  Glycosylated ferritin < 20% |
| **1 point each:**  Age of Onset < 35 years  Arthritis  Sore throat  RES involvement or liver abnormalities  Serositis  Cervical or tarsal ankylosis | **Minor criteria:**  Sore throat  Lymphadenopathy and/or splenomegaly  Liver abnormalities  Negative ANA/RF | **Minor criteria:**  Maculopapular rash  WBC > 10000/ml |

AOSD = adult onset Still's disease; ESR = erythrocyte sedimentation rate; RES = reticuloendothelial system; WBC = white blood count; ANA = antinuclear antibody; RF = rheumatoid factor.

# References

Cush, J.J., Medsger Jr., T.A., Christy, W.C., Herbert, D.C., Cooperstein, L.A. (1987). *Adult-onset Still's disease. Clinical course and outcome.* Arthritis Rheumatol. 30(2):186–194.

Yamagushi, M., Ohta, A., Tsunematsu, T., Kasukawa, R., Mizushima, Y., Kashiwagi, H., et al. (1992). *Preliminary criteria for classification of adult Still’s disease.* J Rheumatol. 19:424–430.

Fautrel, B., Zing, E., Golmard, J.L., Le Moel, G., Bissery, A., Rioux, C., et al. (2002). *Proposal for a new set of classification criteria for adult-onset still disease.* Medicine (Baltimore). 81(3):194-200.
